# Dr. Herbert Davison

**Published:** 1911-09-09

**Authors:** 


Dr. Herbert Davison has died suddenly at Hunstanton. He was in practice in Belvedere, Kent, but had formerly resided in Gosberton, Lincs. He studied at University College, London, and became qualified in 1886 as L.R.C.P.I., later taking the Glasgow diploma.

